# High Prevalence of Metallo-β-Lactamase-Producing *Enterobacter cloacae* From Three Tertiary Hospitals in China

**DOI:** 10.3389/fmicb.2019.01610

**Published:** 2019-08-09

**Authors:** Yimei Cai, Cha Chen, Mei Zhao, Xuegao Yu, Kai Lan, Kang Liao, Penghao Guo, Weizheng Zhang, Xingyan Ma, Yuting He, Jianming Zeng, Liang Chen, Wei Jia, Yi-Wei Tang, Bin Huang

**Affiliations:** ^1^Department of Laboratory Medicine, The First Affiliated Hospital of Sun Yat-sen University, Guangzhou, China; ^2^Department of Laboratory Medicine, The Second Affiliated Hospital of Guangzhou University of Chinese Medicine, Guangzhou, China; ^3^Department of Laboratory Medicine, Guangdong Provincial Hospital of Chinese Medicine, Guangzhou, China; ^4^Department of Laboratory Medicine, Ningxia Hospital of Ningxia Medical University, Yinchuan, China; ^5^Public Health Research Institute Tuberculosis Center, New Jersey Medical School, Rutgers University, Newark, NJ, United States; ^6^Department of Laboratory Medicine, Memorial Sloan Kettering Cancer Center, New York, NY, United States; ^7^Department of Pathology and Laboratory Medicine, Weill Medical College of Cornell University, New York, NY, United States

**Keywords:** carbapenem-resistant, *Enterobacter cloacae*, outbreak investigation, NDM-1, IncX3 plasmids, ST78

## Abstract

*Enterobacter cloacae* has recently emerged as one of the most common carbapenem-resistant *Enterobacteriaceae*. The emergence and spread of metallo-β-lactamase-producing *E. cloacae* have posed an immediate threat globally. Here, we investigated the molecular characteristics of 84 carbapenem-resistant *Enterobacter cloacae* (CREL) collected from three tertiary hospitals in China between 2012 and 2016. Species identification and antimicrobial susceptibility testing were performed using a VITEK-2 system. Carbapenems, polymyxins B, and tigecycline were tested by broth microdilution method. The carbapenem in activation method (CIM) and cefoxitin three-dimensional test were used to detect carbapenemase and AmpC β-lactamase, respectively. Isolates were screened for β-lactam resistance genes by PCR, and expression of *ompC* and *ompF* was determined by qRT-PCR. Genetic relatedness was performed by pulsed-field gel electrophoresis (PFGE) and multilocus sequence typing (MLST), while selected isolates were subjected to whole-genome sequencing. Among the 84 CREL isolates, 50 (59.5%) were detected as carbapenemase producers. NDM-1 was the dominant carbapenemase (80.0%), followed by IMP-26 (8.0%) and IMP-4 (6.0%). Notably, we identified the first NDM-1 and IMP-1 co-producing *E. cloacae*, carrying plasmids of several incompatibility (Inc) groups, including IncHI2, IncHI2A, and IncN. Most isolates showed decreased expression of *ompC* and/or *ompF*, and contained a broad distribution of ESBLs and AmpC β-lactamases. These findings suggested that different molecular mechanisms, including carbapenemase, ESBL and/or AmpC plus loss of porins, have contributed to carbapenem resistance. The *bla*_NDM−1_-harboring plasmids contained highly conserved gene environment around *bla*_NDM−1_ (*bla*_NDM−1_-*ble*_MBL_-*trpF*-*dsbD*-*cutA1*-*groES*-*groEL*), which could be associated with the potential dissemination of *bla*_NDM−1_. IMP-type MBL was located within a variety of integrons and usually contained various gene cassettes encoding multidrug resistance. These isolates produced 54 different pulsotypes, and were classified into 42 STs by MLST. Nineteen *bla*_NDM−1_-positive *E. cloacae* isolates obtained from Ningxia had the same pulsotype (PFGE type 1), belonging to ST78 within clonal complex 74 (CC74). The plasmid-based replicon typing indicated that IncX3 plasmids mediated the dissemination of *bla*_NDM−1_ among these homologous strains. This is the first report on the outbreak of NDM-1-producing *E. cloacae* ST78 with contribution of IncX3 plasmids in Northwestern China. There's an immediate need to intensify surveillance attentively to prevent and control the further spread of NDM-1 in China.

## Introduction

*Enterobacter cloacae*, an opportunistic pathogen ranking the third among all *Enterobacteriaceae* in healthcare-associated infections, may cause various nosocomial infections involving urinary tract, lower respiratory tract, skin and soft tissues, biliary tract, intravenous catheters, and central nervous system (Davin-Regli and Pagès, 2015). *E. cloacae* is intrinsically resistant to ampicillin, amoxicillin/clavulanic, cephamycin, and the 1st and 2nd generation cephalosporins owing to chromosomally encoded AmpC β-lactamase (Jacoby, 2009). Recently, severe comorbid conditions, extensive invasive procedures, and heavy exposure to antibiotics have involved in the global emergence of carbapenem-resistant *E. cloacae* (CREL). Carbapenems have increasingly been used as a common drug for the treatment of nosocomial infections. According to CHINET, one of the largest antimicrobial resistance surveillance networks in China, carbapenem resistance rates among *E. cloacae* were <1.0% in 2007. Whereas, in the year of 2017, the resistance rates to imipenem, meropenem, and ertapenem have rapidly increased to 6.9, 7.0, and 8.2%, respectively. Infections caused by CREL usually result in higher mortality rates, longer hospitalization and higher costs of treatment, which has posed an immediate threat to public health (Kelly et al., 2017).

Resistance to carbapenems is associated with several mechanisms. Carbapenemase, which is largely responsible for carbapenem resistance in *Enterobacteriaceae*, has been classified into three functional groups: class A (mostly KPC, GES), class B metallo-β-lactamase (MBL, mostly IMP, VIM and NDM) as well as class D OXA-type β-lactamase (mostly OXA-48-like) (Tzouvelekis et al., 2012). Genes encoding MBLs are most commonly identified in *E. cloacae* and often carried on mobile genetic elements such as plasmids and transposons. Integrons located on such mobile elements play a crucial role in the horizontal transfer of MBL genes between bacteria (Villa et al., 2014; Lee et al., 2017a). In China, carbapenemase production is largely attributed to MBLs such as VIM-1 (Yang et al., 2014), IMP-4 (Wang et al., 2017), IMP-8 (Yan et al., 2002), and NDM-1 (Dai et al., 2013), among which NDM-1 was even more worrisome for its sharp increase. Since the first case of carbapenem-resistant *E. cloacae* harboring NDM-1 was detected in Chongqing (Wang et al., 2017), NDM-1-producing *E. cloacae* have emerged in various regions across the country (Jia et al., 2018; Jin et al., 2018). Moreover, moderate- to high-level carbapenem resistance in most isolates was closely related to an additional mechanism of resistance, e.g., decreased porin permeability (Majewski et al., 2016). Alteration or loss of non-specific porins, coupled with ESBL and/or AmpC overexpression is considered as one of the main mechanisms of resistance (Wozniak et al., 2012).

However, there has been little epidemiological data on carbapenem-resistant *E. cloacae* (CREL) in certain regions in China, such as Southern (e.g., Guangdong) and Northwestern (e.g., Ningxia) China. To gain insights into the evolution of CREL isolates in these two regions, we conducted a molecular epidemiological study to describe the resistance mechanism to carbapenems, clonal relatedness, and the genetic environment of carbapenemases (NDM-1 or IMP) -encoding plasmids among CREL isolates.

## Materials and Methods

### Bacterial Strains and Characterization

From 2012 to 2016, a total of 84 non-duplicate strains from patients infected by CREL were collected from three tertiary hospitals including hospital A (First Affiliated Hospital of Sun Yat-sen University, Guangdong, China, 25 strains), hospital B (Guangdong Provincial Hospital of Chinese Medicine, Guangdong, China, 18 strains), and hospital C (Ningxia Hospital of Ningxia Medical University, Ningxia, China, 41 strains). Species identification and initial antibiotic susceptibility were analyzed by a VITEK-2 system (bioMerieux, France). Mueller-Hinton broth (Oxoid, UK) supplemented with calcium and magnesium (25.0 mg/L Ca^2+^ and 12.5 mg/L Mg^2+^) was used to test five antibiotics, including imipenem (ApexBio, USA), meropenem (ApexBio, USA), ertapenem (Menlunbio, China), polymyxin B (Sigma, USA), and tigecycline (ApexBio, USA). Minimum inhibitory concentrations (MICs) were performed according to the criteria of the Clinical and Laboratory Standards Institute (CLSI) (M100-S27). Isolates with an MIC of 4 μg/mL were considered resistant to polymyxin B. A susceptibility breakpoint of <2 μg/mL for tigecycline was interpreted in accordance with the European Committee on Antimicrobial Susceptibility Testing (EUCAST) guidelines. Carbapenemase production was assessed by the carbapenem in activation method (CIM) (Aguirre-Quiñonero et al., 2017), while AmpC β-lactamase production was examined by three-dimensional test. Strains used in quality control were *Escherichia coli* ATCC 25922 and *Enterobacter cloacae* ATCC 700323.

### Genotype Analysis

Crude DNA extracts prepared by boiling method were used as template in polymerase chain reactions (PCR). Various β-lactam resistance genes were examined, including carbapenemase genes (*bla*_KPC_, *bla*_NDM_, *bla*_IMP_, *bla*_VIM_, and *bla*_OXA−48_), ESBL-related genes (*bla*_TEM_, *bla*_CTX−M_, and *bla*_SHV_) and AmpC-β-lactamase genes (*bla*_EBC_, *bla*_MOX_, *bla*_CIT_, and *bla*_DHA_). Moreover, *mcr-1* colistin resistance gene and *tetX* tigecycline resistance gene were also determined by using primers as described previously (Pérez-Pérez and Hanson, 2002; Pagani et al., 2003; Biendo et al., 2008; Poirel et al., 2011; Liu et al., 2016). Positive PCR products were sequenced and analyzed by nucleotide homology comparison against GenBank database by BLAST (www.ncbi.nlm.nih.gov/blast/). Details about oligonucleotides and thermal conditions are presented in Supplementary Table 1.

### Outer Membrane Protein Gene Expressions

Expression of genes encoding outer membrane proteins (OmpF and OmpC porins) was determined using real-time reverse transcription PCR. The total RNA was extracted using the Trizol method and reversely transcribed into cDNA using the PrimeScript^TM^ RT reagent Kit with gDNA Eraser (TAKARA, China). Real-time quantitative PCR was performed using SYBR^®^ Green I assay with analysis of dissociation curve (2× SYBR Green q-PCR Master Mix, Biomake, USA) on Applied Biosystems ViiA^TM^ 7 Dx (Life Technologies, USA). Each experiment was performed in triplicate. The expression of porin-encoding genes, *ompF* and *ompC*, relative to *rpoB* was determined using specific primers and conditions described previously (Majewski et al., 2016). Relative expression was derived from the 2-Δ(ΔCT) formula, in which ΔCT represented the difference of cycle threshold (CT) between target genes and *rpoB*, while Δ(ΔCT) represented the difference of ΔCT between CREL strains and a standard strain, *Enterobacter cloacae* ATCC 700323.

### Genetic Homologeity Analysis

All isolates were analyzed by PFGE to determine their genetic relatedness. In brief, the bacterial suspension mixed with equal volumes of low-melting-temperature agarose was cleaved with 1% sodium dodecyl sulfate and proteinase K (Sigma, USA) at 54°C overnight, and then digested with restriction endonuclease *Xba* I (Takara, Dalian, China) at 37°C for 8 h. DNA separation was performed in 0.5× TBE buffer using a pulsed-field electrophoresis system (CHEF MAPPER; Bio-Rad Laboratories, California, USA) under the following conditions: temperature 14°C; voltage 6.0 V/cm; switch angle, 120°; and switch ramp of 2.16–54.17 s for 18 h. Comparison of pulsotypes was performed with Bionumerics software version 6.6. Isolates were allocated into genetic similarity clusters using an 80% cut-off value. MLST was performed as described previously (https://pubmlst.org/ecloacae/). New alleles and sequence types were submitted to the MLST website and approved (https://pubmlst.org/ecloacae/). EnteroBase together with GrapeTree was used to analyze population evolutionary relationship (https://enterobase.readthedocs.io/en/latest/index.html).

### Whole-Genome Sequencing

Six representative strains producing single NDM-1, six strains producing single IMP (according to clonal relatedness), one strain co-producing NDM-1 and IMP-1, and eight strains negative for all β-lactamase genes were selected for whole-genome sequencing. Genomic DNA was extracted using a MiniBEST Bacteria Genomic DNA Extraction Kit (TaKaRa, Dalian, China). DNA library was prepared using a QIAseq FX DNA Library Kit (Qiagen Inc., Valencia, CA) following the manufacturer's recommendations. The quantity and quality of the libraries were assessed with a Qubit dsDNA HS Assay Kit (Life technologies, USA) and LabChip GX Analyzer (Perkin Elmer; Waltham, MA). All barcoded libraries were pooled together in equimolar amounts and sequenced on NextSeq 500 platform (illumina Inc., San Diego, CA). Sequencing raw reads were processed for library adapter removal and filtering using FASTQ preprocessor Fastp v0.12.5 (Chen et al., 2018), followed by *de novo* assembly with SPAdes v3.13.0 (Bankevich et al., 2012) and annotating using Prokka annotation pipeline (Seemann, 2014). Antimicrobial resistance genes and plasmid-typing identification were mined using ABRicate program (https://github.com/tseemann/abricate, v0.8.2), and the contig containing carbapenemase genes, such as *bla*_NDM_ and *bla*_IMP_, were extracted with an in-house Python script, followed by BLAST against NCBI sequence database. The sequence containing *bla*_IMP_ of the variable regions of integrons were analyzed followed by the Integron Database (http://integrall.bio.ua.pt/). Genetic organization analysis was visualized using EasyFig v2.2.3 (Sullivan et al., 2011).

### Statistical Analysis

All the statistical analyses were performed by SPSS 18.0 (IBM Corp., Armonk, USA). The chi-square test was applied to evaluate difference in antibiotic resistance between carbapenemase-positive and carbapenemase-negative subpopulations. Relative changes in gene expression were indicated with median and extremum. All the relevant data were analyzed by non-parametric rank sum test, and was considered statistically significant if *P* < 0.05.

### Ethical Considerations

This study was approved by Institutional Review Board of Second Affiliated Hospital of Soochow University. The study was retrospective and patients were not identified during data collection. Informed consent was not needed for this study.

## Result

### Characteristics of Clinical CREL Isolates

Eighty-four clinical CREL isolates were found to be resistant to at least one carbapenem. These isolates were recovered from different sources: body fluids (*n* = 40), sputum (*n* = 19), urine (*n* = 8), wound secretion (*n* = 5), blood (*n* = 5), pus (*n* = 2), catheter (*n* = 2), cannula (*n* = 1), semen (*n* = 1), and tissue (*n* = 1). These CREL isolates were obtained from patients admitted to the hepatobiliary surgery (*n* = 25, 29.8%), intensive care unit (*n* = 17, 20.2%), general surgery (*n* = 11, 13.1%), neurosurgery (*n* = 7, 8.3%), pediatrics (*n* = 4, 4.8%), burns surgery (*n* = 5, 6.0%), respiratory department (*n* = 3, 3.6%), gastroenterological surgery (*n* = 2, 2.4%), cardiology department (*n* = 2, 2.4%), department of orthopedics (*n* = 2, 2.4%), nephrology department (*n* = 1, 1.2%), and other surgery wards (*n* = 5, 6.0%). Among 84 CREL isolates, 85.7% (72/84) of the isolates were classified as MDR as they were resistant to three or more classes of antibiotics. Seventy-two (85.7%) isolates were resistant to ertapenem, followed by 57 (67.9%) and 54 (64.3%) resistant to imipenem and meropenem, respectively. The strains showed high resistance rates to cefatriaxone (83.3%), ceftazidime (75.0%), cefepime (72.6%), and aztreonam (76.2%), while 43 (51.2%), 51 (60.7%), and 27 (32.1%) strains were resistant to fluoroquinolones, sulfanilamide, and aminoglycosides, respectively. In contrast, 94.0 and 86.9% were susceptible to amikacin and tigecycline. Thirteen *E. cloacae* isolates were resistant to polymyxin B and two isolates were suspected of heteroresistance. Two *E. cloacae* isolates (ECL-ZY07 and ECL-ZY14) with resistance to polymyxin B and tigecycline showed promising *in-vitro* activity against amikacin. Carbapenemase producers presented a dramatically higher resistance rate than negative ones for carbapenemase (*P* < 0.01). Descriptive statistics on antimicrobial susceptibility tests were presented in Tables 1, 2.

**Table 1:** *In vitro* activities of antimicrobial agents against 84 CREL isolates.

**Antibiotic**	**Hospital A (*****n*** **=** **25)**			**Hospital B (*****n*** **=** **18)**			**Hospital C (*****n*** **=** **41)**			**Total (*****n*** **=** **84)**		
	**S%**	**I%**	**R%**	**S%**	**I%**	**R%**	**S%**	**I%**	**R%**	**S%**	**I%**	**R%**
IPM	36.0	20.0	44.0	5.5	66.7	27.8	0.0	0.0	100.0	11.9	20.2	67.9
MEM	36.0	20.0	44.0	83.3	5.6	11.1	0.0	0.0	100.0	28.6	7.1	64.3
ETP	0.0	0.0	100.0	50.0	16.7	33.3	0.0	0.0	100.0	10.7	3.6	85.7
CAO	0.0	4.0	96.0	61.1	11.1	27.8	0.0	0.0	100.0	13.1	3.6	83.3
CAZ	24.0	8.0	68.0	72.2	0.0	27.8	0.0	0.0	100.0	22.6	2.4	75.0
TZP	28.0	0.0	72.0	88.9	0.0	11.1	14.6	2.4	82.9	34.5	1.2	64.3
FEP	24.0	8.0	68.0	83.3	0.0	16.7	0.0	0.0	100.0	25.0	2.4	72.6
ATM	16.0	0.0	84.0	55.6	27.8	16.6	2.4	0.0	97.6	17.8	6.0	76.2
CIP	48.0	4.0	48.0	72.2	0.0	27.8	31.7	7.3	61.0	45.2	4.8	50.0
LEV	56.0	0.0	44.0	72.2	0.0	27.8	29.3	7.3	63.4	46.4	3.6	50.0
GEN	76.0	12.0	12.0	72.2	0.0	27.8	61.0	0.0	39.0	67.8	3.6	28.6
TOB	52.0	24.0	24.0	72.2	0.0	27.8	4.9	73.1	22.0	33.3	42.9	23.8
ATM	96.0	0.0	4.0	88.9	0.0	11.1	95.1	4.9	0.0	94.0	2.4	3.6
SXT	72.0	0.0	28.0	72.2	0.0	27.8	4.9	0.0	95.1	39.3	0.0	60.7
NIT	44.0	44.0	12.0	72.2	16.7	11.1	46.4	34.1	19.5	51.2	33.3	15.5
PB^*^	92.0	0.0	8.0	33.3	0.0	66.7	97.6	0.0	2.4	82.1	0.0	17.9
TGC	88.0	8.0	4.0	94.6	5.6	0.0	82.9	0.0	17.1	86.9	3.6	9.5

S, susceptible; I, intermediate; R, resistant. IPM, imipenem; MEM, meropenem; ETP, ertapenem; CAO, cefatriaxone; CAZ, cefepime; TZP, piperacillin/tazobactam; FEP, cefepime; ATM, aztreonam; CIP, ciprofloxacin; LEV, levofloxacin; GEN, gentamicin; TOB, tobramycin; AMK, amikacin; SXT, sulfamethoxazolen; NIT, furazolidin; PB, polymyxin B; TGC, tigecycline. Hospital A, First Affiliated Hospital of Sun Yat-sen University, Guangdong, China; Hospital B, Guangdong Provincial Hospital of Chinese Medicine, Guangdong, China; Hospital C, Ningxia Hospital of Ningxia Medical University, Ningxia, China. ^*^Two isolates obtained from Hospital B were suspected of heteroresistance to polymyxin B.

**Table 2:** *In vitro* activities of carbapenems to 84 CREL isolates with or without carbapenemase.

**Antibiotics**	**Carbapenemase positive (*****n*** **=** **50)**				**Carbapenemase negative (*****n*** **=** **34)**				^*^P
	**R%**	**MIC (μg/mL)**			**R%**	**MIC (μg/mL)**			
		**MIC range**	**MIC50**	**MIC90**		**MIC range**	**MIC50**	**MIC90**	
IPM	100	8–256	128	256	20.6	1–64	2	4	*P* < 0.01
MEM	100	16–256	128	256	11.8	0.5–8	1	4	*P* < 0.01
ETP	100	32–256	128	256	64.7	0.5–64	2	4	*P* < 0.01

IPM, imipenem; MEM, meropenem; ETP, ertapenem. R, resistant.

* *P-value for comparisons of resistance rates between carbapenemase-positive and carbapenemase-negative groups*.

### Phenotype and Genotype Analysis

Fifty-four isolates were considered positive in the CIM test according to Tijet et al. (2016), but only 50 (59.5%) were demonstrated as carbapenemase producers by PCR analysis. All KPC, NDM, VIM, or IMP producing isolates were unequivocally detected with the CIM test. Four isolates non- producing carbapenemase (one with *bla*_TEM−1_, *bla*_SHV−12_, *bla*_CTX−M_, and *bla*_DHA−1_, three hyperproducing AmpC) were positive by the test. The sensitivity and specificity of the detection assay compared with molecular methods were 100 and 89.5%. The *bla*_NDM−1_ gene was found the most prevalent carbapenemase gene (40/50), followed by *bla*_IMP−26_ (4/50), *bla*_IMP−4_ (3/50), *bla*_IMP−1_ (1/50), *bla*_VIM−4_ (1/50), and *bla*_KPC−2_ (1/50). We observed one *E. cloacae* isolate co-harboring *bla*_NDM−1_ and *bla*_IMP−1_, which resulted in strongly high MICs of all carbapenems (MICs = 256 μg/L). Remarkably, 97.6% (40/41) of the isolates obtained from Ningxia were MBL producers, but only 20.9% (9/43) produced MBL in Guangdong. No carbapenemase gene was detected in hospital B (Guangdong Provincial Hospital of Chinese Medicine, Guangdong, China). Moreover, 83.3% of the isolates continuously produced high AmpC β-lactamase, and 10.7% were pAmpC producers (eight with *bla*_DHA−1_, and one with *bla*_LAP−2_). All carried chromosomal-mediated ACT-type β-lactamase. ESBL producers were found in 36 (42.9%) isolates, among which 25 isolates overproduced AmpC. 19.0% (16/84), 15.5% (13/84), 26.2% (22/84), and 23.8% (20/84) of the isolates were detected as positive for *bla*_TEM−30_, *bla*_TEM−1_, *bla*_CTX−M_, and *bla*_SHV−12_, respectively. Meanwhile, 31.0% (26/84) of the isolates co-expressed carbapenemase and ESBL, and 7.1% (6/84) produced carbapenemase, ESBL and pAmpC simultaneously. None of the isolates were PCR-positive for *bla*_OXA−48_, *mcr-1 or tetX*.

### Expression of *ompC* and *ompF* Genes

Relative expression of *ompF* gene fell within a range of 0.01–1.58, with a median value of 0.12, while expression of *ompC* gene ranged from 0.01 to 36.36, with a median value of 2.80. Thirty-two isolates lost or had lower expression of both major porins, while 52 isolates had decreased expression of one porin, especially for OmpF porin (50/52). Isolates with a combination of carbapenemase and porin loss showed extensively high carbapenem MICs. All 34 non-carbapenemase-producing isolates had lost at least one porin, among which 22 isolates had reduced expression of both major porins. Additionally, 7 isolates produced both ESBL and AmpC, and 24 isolates produced only one type of enzyme. The remaining 3 isolates were negative for ESBLs (TEM, SHV, or CTX-M) and AmpC. Porin loss together with AmpC overexpression typically resulted in insensitivity to one or two carbapenems. Compared with isolates non-susceptible to three carbapenems and isolates only non-susceptible to one or two carbapenems, the latter had significantly lower expression of *ompC* (*P* < 0.01), but there was no difference in *ompF* expression among two groups (*P* = 0.398). Further, lower expression of *ompC* was significantly associated with increasing MIC of imipenem (*P* < 0.01). All details about antibiotic susceptibilities and porin expression of CREL isolates were given in Table 3.

**Table 3:** Drug-resistance characteristics of 84 CREL isolates.

**Isolate**	**MIC (μg/L)**			**Carbapenemase**	**ESBL**	**AmpC**	**OMPF**	**OMPC**
	**IPM**	**MEM**	**ETP**					
ECL-ZY01	64	64	64	NDM-1	–	+	0.19↓	1.56↑
ECL-ZY02	64	64	64	NDM-1	SHV-12	+	0.07↓	0.13↓
ECL-ZY03	1	1	4	–	CTX-M; TEM-30	+	0.10↓	2.07↑
ECL-ZY04	1	1	2	–	CTX-M; TEM-30	–	0.13↓	1.34↑
ECL-ZY05	1	1	4	–	–	+	0.02↓	0.02↓
ECL-ZY06	8	8	64	–	CTX-M; TEM-1; SHV-12	–	0.01↓	1.36↑
ECL-ZY07	1	1	4	–	CTX-M; TEM-30	+	0.28↓	0.58↓
ECL-ZY08	256	256	256	NDM-1	CTX-M; TEM-1; SHV-12	+	0.26↓	0.66↓
ECL-ZY09	128	128	128	NDM-1	CTX-M; TEM-30; SHV-12	+	0.31↓	3.31↑
ECL-ZY10	2	2	4	–	–	+	0.01↓	0.01↓
ECL-ZY11	32	32	128	IMP-8	–	+	0.01↓	0.02↓
ECL-ZY12	1	1	4	–	–	+	0.29↓	3.69↑
ECL-ZY13	256	256	256	NDM-1	TEM-1; SHV-12	+	0.54↓	0.63↓
ECL-ZY14	2	2	4	–	SHV-12	+	0.01↓	0.82↓
ECL-ZY15	1	1	2	–	–	+	0.28↓	0.74↓
ECL-ZY16	2	2	8	–	CTX-M	+	0.01↓	0.21↓
ECL-ZY17	2	2	2	–	CTX-M; TEM-1	+	0.19↓	2.76↑
ECL-ZY18	128	128	128	IMP-4	CTX-M; TEM-1	+	0.01↓	0.09↓
ECL-ZY19	1	1	4	–	–	+	0.06↓	6.48↑
ECL-ZY20	1	1	2	–	–	+	0.22↓	7.52↑
ECL-ZY21	1	1	2	–	–	+	0.19↓	5.41↑
ECL-ZY22	256	256	256	KPC-2	CTX-M; TEM-1; SHV-12	+	0.01↓	0.01↓
ECL-ZY23	8	16	64	VIM-4		+	0.01↓	2.70↑
ECL-ZY24	256	256	256	NDM-1	TEM-1; SHV-12	+	0.20↓	0.47↓
ECL-ZY25	2	2	4	–	CTX-M	+	0.74↓	0.01↓
ECL-NX01	128	128	128	NDM-1	–	+	0.61↓	10.92↑
ECL-NX02	64	64	32	NDM-1	CTX-M; TEM-1	+	0.38↓	36.36↑
ECL-NX03	128	128	128	NDM-1	–	+	0.23↓	11.28↑
ECL-NX04	128	128	128	NDM-1	–	+	0.32↓	13.81↑
ECL-NX05	32	32	32	IMP-26	TEM-30; SHV-12	+	0.65↓	6.99↑
ECL-NX06	256	256	256	NDM-1	CTX-M; TEM-30	–	0.12↓	7.07↑
ECL-NX07	32	32	32	IMP-26	TEM-30; SHV-12	+	0.01↓	6.06↑
ECL-NX08	256	256	256	NDM-1	CTX-M; TEM-30	–	0.09↓	0.01↓
ECL-NX09	128	128	128	NDM-1	–	+	0.08↓	12.44↑
ECL-NX10	128	128	128	NDM-1	–	+	0.20↓	8.37↑
ECL-NX11	128	128	128	NDM-1	–	+	0.22↓	10.12↑
ECL-NX12	128	128	128	NDM-1	–	+	0.14↓	7.62↑
ECL-NX13	128	128	128	NDM-1	–	+	0.15↓	9.35↑
ECL-NX14	128	128	128	NDM-1	–	+	0.16↓	10.11↑
ECL-NX15	128	128	256	NDM-1	–	+	0.15↓	9.91↑
ECL-NX16	128	128	128	NDM-1	–	+	0.22↓	11.3↑
ECL-NX17	256	256	256	NDM-1	CTX-M; TEM-30	–	0.11↓	8.41↑
ECL-NX18	64	8	64	–	CTX-M; TEM-1; SHV-12	+	0.06↓	0.49↓
ECL-NX19	128	128	128	IMP-26	TEM-1; SHV-12	+	0.15↓	6.92↑
ECL-NX20	256	256	256	NDM-1	SHV-12	+	0.12↓	5.83↑
ECL-NX21	128	128	128	NDM-1	–	+	0.14↓	10.05↑
ECL-NX22	128	128	128	NDM-1	–	+	0.14↓	9.20↑
ECL-NX23	128	128	128	NDM-1	–	+	0.09↓	12.28↑
ECL-NX24	32	32	32	IMP-4	SHV-12	+	1.58↓	13.48↑
ECL-NX25	256	256	256	NDM-1	CTX-M; TEM-30	–	0.03↓	10.59↑
ECL-NX26	128	128	128	NDM-1		+	0.12↓	15.44↑
ECL-NX27	32	32	64	IMP-4	CTX-M; SHV-12	+	0.06↓	6.73↑
ECL-NX28	256	256	256	NDM-1	CTX-M; TEM-30	–	0.09↓	0.01↓
ECL-NX29	256	256	256	NDM-1	CTX-M; TEM-30	–	0.09↓	0.05↓
ECL-NX30	128	128	128	NDM-1	–	+	0.15↓	8.98↑
ECL-NX31	128	128	128	NDM-1	–	+	0.19↓	6.43↑
ECL-NX32	128	128	128	NDM-1	–	+	0.16↓	8.14↑
ECL-NX33	128	128	128	NDM-1	–	+	0.08↓	7.85↑
ECL-NX34	128	128	128	NDM-1	TEM-30	+	0.07↓	4.04↑
ECL-NX35	256	256	256	NDM-1 IMP-1	TEM-30; SHV-12; LAP-2	+	0.01↓	6.31↑
ECL-NX36	128	128	128	NDM-1	–	+	0.17↓	3.06↑
ECL-NX37	128	128	128	IMP-26	TEM-30; SHV-12	+	0.16↓	3.06↑
ECL-NX38	128	128	128	NDM-1	–	+	0.07↓	2.79↑
ECL-NX39	64	64	64	NDM-1	CTX-M; TEM-30; SHV-12	–	0.23↓	5.66↑
ECL-NX40	64	64	64	NDM-1	CTX-M; TEM-1; SHV-12	–	0.09↓	2.81↑
ECL-NX41	128	128	128	NDM-1	TEM-1; SHV-12	+	0.12↓	1.29↑
ECL-SZY01	2	0.5	0.5	–	–	+	0.01↓	0.01↓
ECL-SZY02	2	0.5	0.5	–	–	–	0.01↓	0.01↓
ECL-SZY03	4	0.5	0.5	–	–	+	0.01↓	0.01↓
ECL-SZY04	4	4	4	–	–	+	0.01↓	0.01↓
ECL-SZY05	2	0.5	0.5	–	–	+	0.01↓	0.02↓
ECL-SZY06	2	0.5	0.5	–	–	+	1.35↑	0.01↓
ECL-SZY07	2	0.5	0.5	–	–	+	0.06↓	0.01↓
ECL-SZY08	4	1	1	–	–	+	0.01↓	0.01↓
ECL-SZY09	2	0.5	1	–	–	+	0.01↓	0.01↓
ECL-SZY10	2	0.5	0.5	–	–	+	0.01↓	0.20↓
ECL-SZY11	2	0.5	0.5	–	–	+	0.27↓	0.82↓
ECL-SZY12	2	0.5	0.5	–	–	+	0.01↓	0.01↓
ECL-SZY13	1	1	4	–	–	–	0.90↓	0.01↓
ECL-SZY14	4	1	1	–	–	+	1.20↑	0.01↓
ECL-SZY15	2	2	4	–	–	+	0.03↓	0.01↓
ECL-SZY16	4	4	2	–	TEM-1	–	0.01↓	4.5↑
ECL-SZY17	2	1	4	–	–	–	0.01↓	3.91↑
ECL-SZY18	2	1	4	–	–	+	0.01↓	0.08↓

*IPM, imipenem; MEM, meropenem; ETP, ertapenem. Arrows indicated the relative variation of expression in ompC and ompF genes. AmpC β-lactamase was examined by cefoxitin in three-dimensional test. The plus and minus were used to describe the positive or negative results of β-lactamases. Arrows represents up- and down- regulation of OM expression*.

### PFGE Analysis

**Figure 1:**
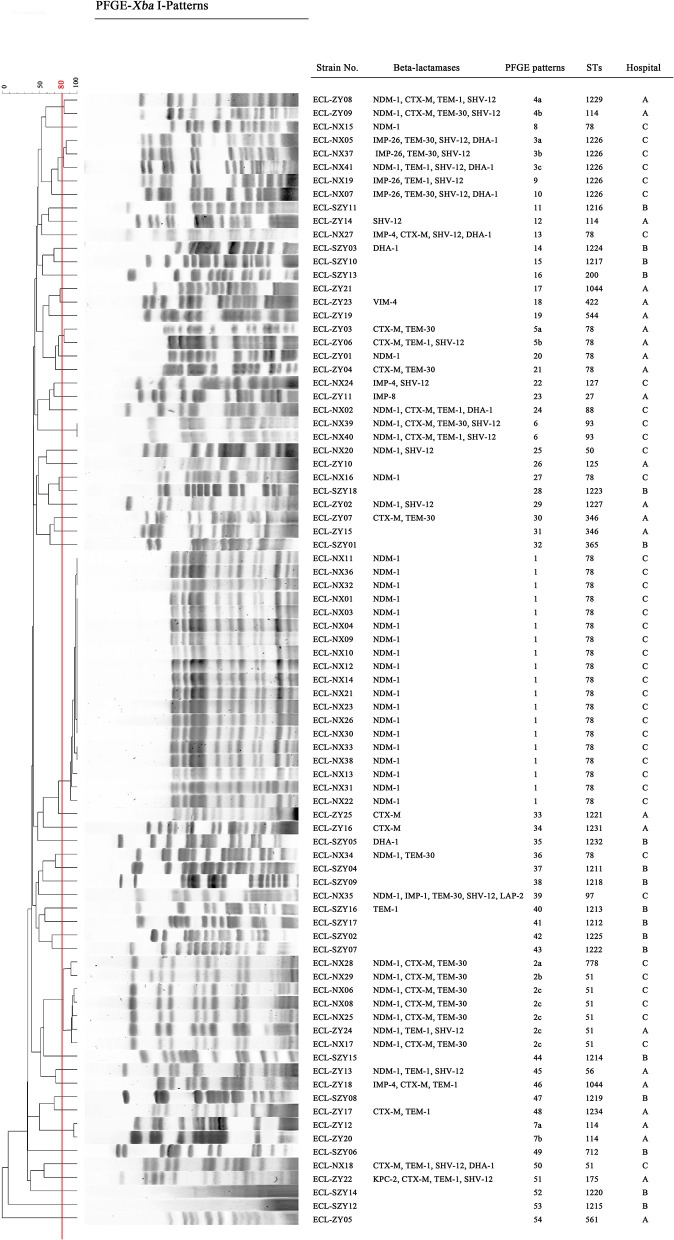
Dendrogram of the PFGE profiles of CREL isolates. The Dice coefficient was used to identify different types with a cutoff of 80.0% similarity. Hospital A, First Affiliated Hospital of Sun Yat-sen University, Guangdong, China; Hospital B, Guangdong Provincial Hospital of Chinese Medicine, Guangdong, China; Hospital C, Ningxia Hospital of Ningxia Medical University, Ningxia, China.

**Figure 2:**
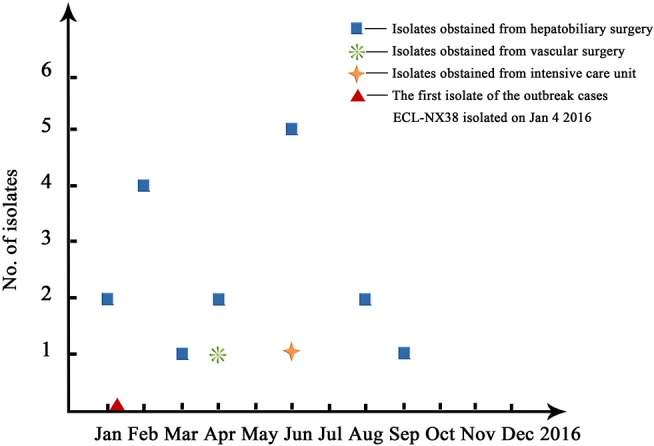
An overall time distribution of predominated ST78 clones in Hospital C in the year of 2016.

Pulsed-field gel electrophoresis patterns of the 84 CREL isolates were identified into 54 pulsotypes (clusters 1–54), and of these, PFGE types 2, and 3 could be classified into 3 subtypes, while types 4, 5, and 7 were classified into 2 subtypes (Figure 1). PFGE revealed genetic diversity, whereas PFGE types 1, 2, 3, 4, 5, 6, and 7 were observed in 19, 6, 3, 2, 2, 2, and 2 different patients, respectively. Notably, all the 19 strains with a single dominant pulsotype (PFGE type 1) were isolated from patients in hepatobiliary surgery (17/19), intensive care unit (1/19), and vascular surgery (1/19) during a detection peak period (from Jan. 2016 to Sep. 2016) in hospital C. As shown in Figure 2, the strain ECL-NX38 which was isolated from a 78-year-old patient in hepatobiliary surgery on January 4, 2016 could be the first strain of these outbreak cases. Besides, three strains of type 3 were isolated from patients in two different wards in hospital C during 1-year period. All the six strains of PFGE type 2 and two strains of type 6 were distributed sporadically in different wards in hospital C. All details about clinical characteristics and PFGE patterns of 41 CREL isolates from hospital C was shown in Table 4. However, all pulsotypes were single isolates in hospital B. Two strains of PFGE type 4 were isolated from patients in ICU in hospital A within 1 year; two strains of type 5 were isolated from patients in two different wards within 2 months; while two strains of type 7 were isolated from patients in hepatobiliary surgery within 1 month (data not shown).

**Table 4:** PFGE patterns and clinical characteristics of 41 CREL isolates obtained from Hospital C.

**Pulsotypes**	**Strain no**.	**Departments (*n*)**	**Specimens (*n*)**	**Years (*****n*****)**				
				**2012**	**2013**	**2014**	**2015**	**2016**
		Hepatobiliary surgery (17)						19
1	19	Intensive care unit (1),	bi (17), su (1), pf (1)					
		Vascular Surgery (1)						
2a	1	Intensive care unit (1)	ur (1)		1			
2b	1	Intensive care unit (1)	pf (1)	1				
		Neurosurgery (1)						
2c	4	Cardiology department (1)	sp (2), ur (1), su (1)	1		2	1	
		Respiratory Medicine (1)						
		Intensive care unit (1)						
3a	1	Burns surgery (1)	se (1)					1
3b	1	Burns surgery (1)	se (1)					1
3c	1	Hepatobiliary surgery (1)	bl (1)				1	
6	2	Hepatobiliary surgery (1)	bi (1), sp (1)		1			1
		Intensive care unit (1)						
8	1	Vascular surgery (1)	su (1)					1
9	1	Burns surgery (1)	se (1)					1
10	1	Burns surgery (1)	ps (1)		1			
13	1	Burns surgery (1)	ca (1)					1
22	1	General surgery	sp (1)					1
24	1	Oral surgery (1)	sb (1)		1			
25	1	PICU (1)	ca (1)				1	
27	1	Orthopedics (1)	ps (1)					1
36	1	Hepatobiliary surgery (1)	bi (1)					1
39	1	Cardiology department (1)	pf (1)					1
50	1	Pediatrics	bl (1)					1
Total	41			2	4	2	3	30

*bi, bile; su, drainage; sp, sputum; ur, urine; se, secretion; ps, pus; ca, catheter; bl, blood*.

### MLST Analysis

**Figure 3:**
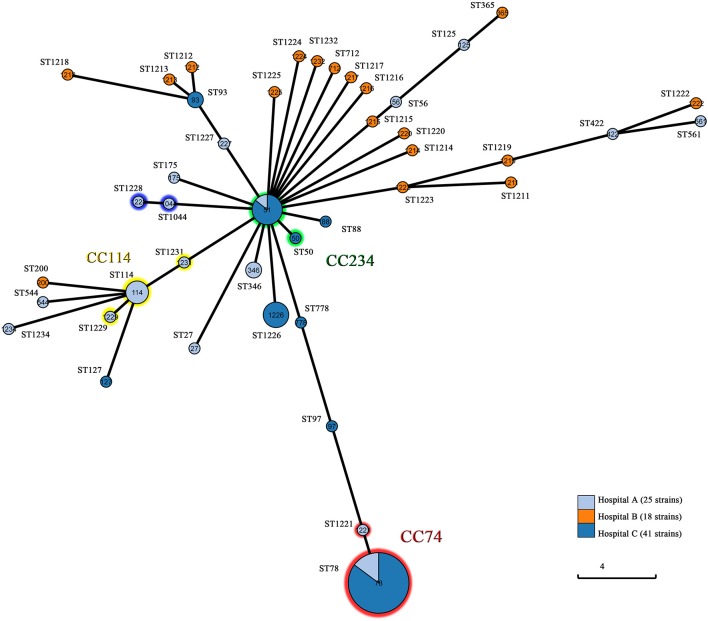
Population structure of the 84 CREL isolates by GrapeTree analysis. Each circle is a Node and each line is a branch. Node size is dependent on the number of strains within that node. Numbers in circles represent ST types. Branch length varies on the distance between nodes. Hospital A, First Affiliated Hospital of Sun Yat-sen University, Guangdong, China; Hospital B, Guangdong Provincial Hospital of Chinese Medicine, Guangdong, China; Hospital C, Ningxia Hospital of Ningxia Medical University, Ningxia, China.

MLST distinguished 42 STs including 22 novel STs (ST1211-ST1229, ST1231, ST1232, and ST1234). The most prevalent ST was ST78 (*n* = 27, 32.1%), followed by ST51 (*n* = 7, 8.3%), ST1226 (*n* = 5, 6.0%), ST114 (*n* = 4, 4.8%), ST93 (*n* = 2, 2.4%), ST346 (*n* = 2, 2.4%), and ST1044 (*n* = 2, 2.4%). The remaining STs contained one isolate for each. MLST showed subtype diversity, which was relatively consistent with PFGE. As expected, PFGE was more discriminatory than MLST. Most isolates belonging to different subtypes of the same pulsotype had an identical ST. The only exceptions were one single-locus variant pairs (SLVs) (ST114 and ST1229) and five SLVs (ST51 and ST778), among which isolates of one ST were clustered into one of the pulsotypes of the other ST. Of the 42 STs, 33 (78.6%) were singletons, 6 (14.3%) had single SLVs and 3 (7.1%) clustered into a bigger group within CC114. The ST114-centered group encompassed 6 isolates of 3 STs including ST114 (*n* = 4), ST1229 (*n* = 1), and ST1231 (*n* = 1), and isolates were all obtained from hospital A. Twenty-eight isolates from hospital A and C belonged to ST78 (*n* = 27) and ST1221 (*n* = 1) within CC74, while eight isolates represented ST51 (*n* = 7) and ST50 (*n* = 1) within CC234. Other single SLVs consisted of ST1044 and ST1228, indicating direct clonal evolution between the SLVs. All of the STs were analyzed by Grapetree in order to reveal their relationships (Figure 3). The ST78 clones of eight PFGE pulsotypes exhibited various β-lactamase profiles. The most prevailing type harboring NDM-1 belonged to ST78 and carried plasmids of two or three Inc types, including IncFIB, IncX3, and IncX4 (Supplementary Table 2). Plasmid-based replicon typing revealed that the IncX3 type epidemic plasmid carrying *bla*_NDM−1_ caused an outbreak of *E. cloacae*. These dominated clones carried several other β-lactamase genes, including *bla*_ACT−5_ and *bla*_OXA−1_. However, ST78 *E.cloacae* isolates were not always associated with NDM-1. Three ST78 strains from hospital A produced different ESBLs but not NDM-1, and one ST78 *E.cloacae* from hospital C produced IMP-4. Each of these comprised various Inc types of plasmids. Six ST51 isolates with highly similar pulsotypes produced NDM-1-type MBL, and four isolates belonged to ST1226 diversified into three pulsotypes, were frequently related to IMP-26. Due to the deficiency of clinic case and limited data, all these observations were still to be further validated.

### Genetic Environments of NDM-1 or IMP Carrying Contigs

**Figure 4:**
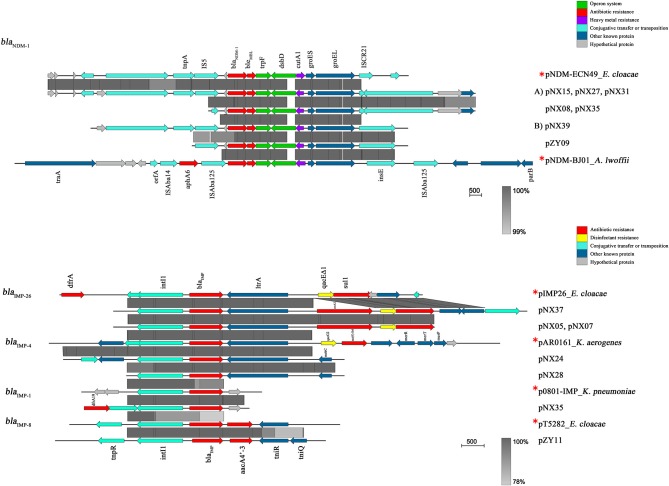
Linear comparison of the representative plasmid sequences carrying NDM-1 or IMP. ^*^Reference plasmids were as following: pNDM-1-ECN49 in *E. cloacae* (GenBank Accession No. KP765744), pNDM-BJ01 in *A. lwoffii* (GenBank Accession No. JQ001791), pIMP26 in *E. cloacae* (GenBank Accession No. MH399264), pAR0161 in *K. aerogenes* (GenBank Accession No. MF344574), p0801-IMP in *K. pneumoniae* (GenBank Accession No. KT345947), and pT5282 in *E. cloacae* (GenBank Accession No. MF344574). Block arrows indicate confirmed or putative open reading frames (ORFs) and their orientations. Arrow size is proportional to the predicted ORF length. Regions of homology are marked by gray shading. The color code is as follows: red, antibiotic resistance genes; purple, heavy metal resistance genes; yellow, disinfectant-resistance genes; green, operon; cyan, genes associated with the transfer; dark blue, other known genes; hypothetical and unknown genes are represented by light-gray arrows.

As shown in Figure 4, seven NDM-1-harboring plasmids had a common genetic structure, which was highly similar to some previous plasmids, such as pNDM-ECN49 (GenBank Accession No. KP765744). Compared with another epidemic plasmid pNDM-BJ01 in *A. lwoffii* (GenBank Accession No. JQ001791), however, a complete or partial IS*5* supplanted the IS*Aba125* upstream of the *bla*_NDM−1_ gene. The *bla*_NDM−1_-like-carrying plasmids could be divided into two different types. Type A plasmids mainly differed by the insertion of a Tn*3*-like transposon downstream of *groES*. The Tn*3*-like transposon consisted of *tnpA, tnpR*, and two ORFs encoding hypothetical proteins, flanked by a pair of 38 bp inverted repeats (IR). The inverted repeat motifs of target site 6 bp at the boundaries of the Tn*3*-like element indicated insertion by transposition. However, IS*91*-like element, which probably transpose via a rolling-circle replication mechanism, was located downstream of *groES* among two plasmids of Type B.

All of the *bla*_IMP_ genes were situated within class 1 integrons. The *bla*_IMP−8_ gene was identified in In655 containing *bla*_IMP−8_-*aacA4*′*-3*, which was previously detected in *E. cloacae* (GenBank Accession No. MF344574). Three genetic mutations in coding region of *intl1* (A38978C, G38958C, and T38953C) were observed in the integron mediated IMP-8. The integron harboring *bla*_IMP−26_ contained the gene cassette array: *bla*_IMP−26_-*ereA2*–*qacE*Δ*1*/*sul1*, and was detected in 3 isolates. The integron had a structurally similar cassette to pIMP26 in *E. cloacae* (GenBank Accession No. MH399264), but the difference in *ereA2* gene insertion resulted in erythromycin resistance. Integrons of *bla*_IMP−4_ gene had a similar genetic structure to pAR0161 (GenBank Accession No. MF344574), and 3′-CS *qacE*Δ*1*-*sul1* were also observed downstream. However, we were unable to sequence the complete integron-related gene cassettes due to the limitations of short-read sequencing. The *bla*_IMP−1_ gene was determined in a shorter contig accompanied with a partial *aacA4* gene downstream. The sequence alignment revealed that one synonymous mutation (T39C) were observed, compared with the widely reported sequence of *bla*_IMP−1_ gene (GenBank Accession No. MG287118). This resultant sequence was almost completely consistent with the partial published sequence in *K. oxytoca* p7121-IMP (GenBank Accession No. KX784502.1).

## Discussion

*E. cloacae* is frequently implicated in serious healthcare-associated infections, with a high proportion of carbapenemase producers among carbapenem-resistant *Enterobacteriaceae* (Davin-Regli and Pagès, 2015). *E. cloacae* has been reported to acquire carbapenemases including KPC-2 (Andrade et al., 2018), NDM-1 (Torres-González et al., 2015), IMP-4 (Lee et al., 2017b), IMP-26 (Dai et al., 2013), IMP-8 (Pang et al., 2016), IMP-1 (Aoki et al., 2018), and VIM-4 (Sonnevend et al., 2017). Our study demonstrated the high prevalence of carbapenemase among carbapenem-resistant *E. cloacae* (CREL) in south and northwest of China (59.5%). MBLs, especially NDM-1 was, the dominant mechanism of resistance to carbapenems in *E. cloacae* in China. Most cases of infections or colonizations of NDM-1-producing bacteria originated in southern Asia, most commonly in India (Walsh and Toleman, 2011). The different prevalence of NDM-1 between countries might be explained by its high genetic transfer rate among unrelated bacterial species. Meanwhile, IMP-type MBL has been identified as the second common carbapenemase (10.7%). Compared to the worldwide distribution of IMP-4 (Leung et al., 2013; Lee et al., 2017b) and IMP-8 (Wang et al., 2015; Pang et al., 2016), IMP-26 and IMP-1 were also detected in our study. Although KPC or VIM-producing *E. cloacae* has been reported to spread rapidly in the last decade (Kanamori et al., 2017; Sonnevend et al., 2017; Daniels et al., 2018), the prevalence remained relatively low in our study. Moreover, the carriage rate of ESBL-related genes among CREL isolates was high (42.9%), though no dominant ESBL type was identified. Our study showed that 83.3% of the isolates continuously produced high AmpC β-lactamase. ACT-type inducible AmpC enzyme remained conservative. DHA-type, however which has become the most prevalent plasmid-mediated AmpC β-lactamase, was less frequently described in the literature with regard to *E. cloacae*. These additional β-lactamases might explain a slight increase of carbapenem resistance among CREL isolates (Souna et al., 2014). Of note, two MBLs, NDM-1 and IMP-1, were firstly identified to be co-expressed in *E. cloacae*, which was isolated from a patient after cardiac surgery in Ningxia. This XDR *E. cloacae* isolate co-harbored *bla*_SHV−12_, *bla*_TEM−30_, *bla*_LAP−2_, *bla*_ACT−5_, *qnrS1, aac(6')-IIc, aadA6, aph(3')-Ia, aph(6)-Id, catA2, sul1*, and *dfrA10*. Resistance genes existed in multiple incompatibility groups, including IncN, IncHI2A, and IncHI2, which could further aggravate the dissemination under the selective pressure of antibiotics (Supplementary Table 2).

Besides, the absence or reduced expression of porins coupled with ESBL and/or AmpC overexpression, could lead to increased carbapenem MICs (Babouee Flury et al., 2016). In our study, most isolates had decreased membrane permeability caused by low expression of *ompC* or *ompF*, or both. Most isolates exhibited down-regulation of *ompF*, but up-regulation of *ompC*. These results were consistent with the previous reports and indicated that the decreased expression of *ompF* might lead to the potential role of OmpC-directed OM protein polarization (Majewski et al., 2016). Combinations of either ESBL or AmpC and porin loss played a pivotal role especially among carbapenemase-negative isolates. These isolates had lost at least one porin, among which 91.2% (31/34) of the ioslates produced different types of β-lactamase. Analysis of *ompC* expression showed a significant decrease among isolates only non-susceptible to imipenem. The decrease or loss of OmpC, a smaller pore size membrane porin compared to that of OmpF, might be correlated with the slightly increase in imipenem MIC (Lavigne et al., 2012). Hence, porin loss combined with ESBL and/or AmpC overexpression was one of the major mechanisms of drug resistance. Carbapenemase, ESBL and/or AmpC hyperproduction plus porin loss usually resulted in extensively high carbapenem MICs.

In this study, we observed significantly differential distribution and molecular characteristics of CREL isolates in two geological regions. In Ningxia, China, MBLs, especially NDM-1, was identified in the highest proportion, indicating a great potential risk of spread of drug-resistant strains in northwest of China. However, lower prevalence of carbapenemase producers has been described in Guangdong, China during our investigation, and that approximately half of isolates showed intermediate resistance to one or two carbapenems. This phenomenon might be mainly due to additional β-lactamases and impaired permeability. No carbapenemase producer was found in hospital B (Guangdong Provincial Hospital of Chinese Medicine, Guangdong, China). The possible interpretation was that carbapenems might be utilized less frequently in these hospitals. In most cases, patients with infections caused by CREL usually suffer from critical basic diseases, lower immune function, and all kinds of invasive operations, and they may be less inclined to TCM hospitals. Another explanation, probably different genetic events among *E. cloacae* isolates have occurred over time in TCM hospitals and carbapenemase genes was very rare or unique. Besides, about other mechanisms, especially high level of AmpC β-lactamases plus porin loss, and overexpression of efflux pumps, would play a more important part in carbapenem resistance among these *E. cloacae* isolates. Therefore, a variety of mechanisms could induce resistance in varying degrees in the process of drug resistance in *E. cloacae*.

The most susceptible antimicrobials to CREL isolates was amikacin (94.0%), followed by tigecycline (86.9%), and polymyxin B (82.1%). Amikacin, an aminoglycoside group antibiotic, has been restricted in recent years due to its side effects such as nephrotoxicity and ototoxicity (Wargo and Edwards, 2014). Polymyxin B and tigecycline are currently the most active therapeutic option for carbapenem-resistant *Enterobacteriaceae* including CREL (Rafailidis and Falagas, 2014). However, polymyxin B and tigecycline resistance has sporadically occurred in recent years (Kumar, 2016; Karaiskos et al., 2017). We found 13 isolates resistant to polymyxin B and 11 isolates non-susceptible to tigecycline, but *mcr-1* and *tetX* were not detected. Resistance to polymyxins might be associated with alterations in the lipopolysaccharide structure and overexpression of efflux pumps (Lim et al., 2010). Besides, ribosome protection and excessive expression of efflux pumps might contribute to decreased susceptibility to tigecycline (Pournaras et al., 2016). Remarkably, polymyxin B heterogeneous subpopulations were detected in two isolates, indicating that more consideration should be taken to heterogeneously colistin resistant *E. cloacae*.

Our results suggested that NDM-1-harboring plasmids contained a highly conserved region around *bla*_NDM−1_ (*bla*_NDM−1_-*ble*_MBL_-*trpF*-*dsbD*-*cutA1*-*groES*-*groEL*), which might be involved in the further spread of *bla*_NDM−1_. The highly similar genetic structure was also obtained from various NDM-1-encoding plasmids, such as *C. freudii* from China (Yang et al., 2015) and *K. pneumoniae* from Australia (Wailan et al., 2016). More importantly, the region flanking *bla*_NDM−1_ was highly homologous to some *Acinetobacter* spp. isolated from China (Bogaerts et al., 2013). Recent studies proposed that the acquisition of *bla*_NDM−1_ in *Enterobacteriaceae* might be derived from *Acinetobacter* spp. via horizontal transmission of drug-resistant plasmids (Bogaerts et al., 2013). These findings suggested that the potential dissemination of acquired *bla*_NDM−1_ gene among different bacterial species had become an emerging global threat. IMP-type MBL was located within a variety of integrons. Similar to previous studies, IMP-producing *E. cloacae* presented a diverse genomic environment due to the transfer and rearrangement of plasmids and integrons in all probability (Aoki et al., 2018). Different genetic events might occur among *E. cloacae* isolates and other isolates, leading to the diversity of integrons in our study. The presence of various integrons associated with plasmids could facilitate horizontal or clonal transmission among bacteria of different genera and species through conjugation.

PFGE revealed that 84 CREL isolates possessed 54 pulsotypes. Nineteen NDM-1-producing *E. cloacae* isolates obtained from Ningxia had a single dominant pulsotype. These predominated clones have obtained fast transmission in hepatobiliary surgery, vascular surgery, and ICU during a peak period (from Jan. 2016 to Sep. 2016), which might be the evidence of intra-hospital clonal dissemination. The further examining revealed that all outbreak strains were ST78, belonging to clonal complex CC74, which was considered as the most versatile genetic lineages among the world (Izdebski et al., 2015). The uniform genetic background among NDM-1-producing *E. cloacae* with conjugative IncX3 plasmids showed horizontal transmission between epidemic isolates. These results suggested that IncX3-type plasmids might contribute significantly to the outbreak of *bla*_NDM−1_ within *E. cloacae* in China. Previous studies have reported that the IncX3-type plasmid was a serious threat, particularly because of the global spread of NDM-1-producing *Enterobacteriaceae* spp (Yang et al., 2015). The outbreak strains producing NDM-1 co-harbored multiple resistance genes, including *bla*_ACT−5_, *bla*_OXA−1_, *sul1, dfrA15b, mphA, catB3, arr3* as well as the *qnrA1* and *aac(6')Ib-cr* genes encoding quinolone resistance. Studies have reported the emergency and spread of *E. cloacae* ST120, ST74, ST418, and ST88 producing NDM-1 in China (Liu et al., 2015; Jia et al., 2018; Jin et al., 2018). This implied that the *bla*_NDM−1_ gene might have been transmitted to multiple clones of *E. cloacae* in many parts of China. To the extent of our knowledge, this is the first identification of an outbreak of *bla*_NDM−1_ harboring *E. cloacae* ST78 isolates in China. Our study suggested that ST78 has become a successful hospital-related clone with a unique ability to accept plasmids for further dissemination in China. ST78 was one of the leading clonal lineages with increased epidemic potential, which might be associated with the spread of carbapenem resistance (Miyoshi-Akiyama et al., 2013). In summary, we reported that CREL isolates were still distributed popularly in Guangdong, China during our investigation. However, we emphasized the spread of NDM-1-producing *E. cloacae* ST78 with contribution of IncX3 plasmids in Ningxia, China. The outbreak has provided a new model for the dissemination of the *bla*_NDM−1_-haboring *E. cloacae* in China. Therefore, epidemiological surveillance of resistance is vital and should be performed routinely, especially in high-risk departments.

MLST distinguished 42 STs with ST78 (32.1%), ST51 (8.3%), ST1226 (6.0%), and ST114 (4.8%), and being the predominant STs. Previous studies revealed that ST66, ST78, ST108, and ST114 were the most prevalent and widespread *E. cloacae* STs (Izdebski et al., 2015). Our study showed that 36.9% (31/84) of the isolates belonged to the potentially high-risk international clones, with ST78 prevailing. We found that *E. cloacae* ST78 producing NDM-1 with a higher epidemic potential might be more prone to cause severe drug-resistant outbreaks. The ST78 clones demonstrated various β-lactamase profiles. Early studies also indicated a lack of strict correlation between β-lactamase profiles and ST78. The ST78 *E. cloacae*, originally identified in Japan, was a major international clone. The ST78 clones was widely distributed and independently obtained plasmids with β-lactamase genes, making specific pulsotypes more suitable for further spread (Miyoshi-Akiyama et al., 2013). In addition, seven ST51 isolates belonged to CC234, one of dominant clonal groups with high prevalence and wide distribution. Four ST114 isolates were diversified into multiple pulsotypes and caused some sporadic isolates. As the central genotype of CC114, ST114 has a wide global distribution formerly detected in Japan, France, Italy, Spain, Greece and Israel, mainly associated with *bla*_CTX−M−15_ (Izdebski et al., 2015). Moreover, we identified a novel ST type, ST1226 detected in 5 isolates with highly similar pulsotypes, and might be related to the carriage of *bla*_IMP−26_ in our study. Strikingly, we described in detail one *E. cloacae* ST97, which co-harbored *bla*_NDM−1_ and *bla*_IMP−1_ as well as multiple resistance genes.

Compared with carbapenem-resistant *K. pneumoniae* (CRKP), our study demonstrated that NDM-1-type MBL was the most commonly identified in *E. cloacae*. Recent surveillance data in China indicated that NDM was predominant in *E. coli* and *E. cloacae*, whereas KPC appeared to be most common in *K. pneumoniae* (Wang et al., 2018). Most previous studies revealed that the substantial burden of KPC-type carbapenemase mainly lied in North America, Latin America and Europe (Munoz-Price et al., 2013). However, NDM-1-mediated carbapenem resistance was widespread in Asia, such as India, Pakistan, and China (Nordmann et al., 2012). In contrast to the close connection between ST258/ST11 and *bla*_KPC_ in *K. pneumoniae* (Liu et al., 2018), *bla*_NDM−1_ apparently has a higher association with IncX3-type plasmids regardless of ST type in *E. cloacae*. Diverse clones of NDM-1-carrying *E. cloacae* have been widely distributed in geography. Evidence showed that clonal expansion was the cause of most transmitted cases. Thus the rapid spread of NDM-1-producing *E. cloacae* has comprised one of the greatest challenges to global health.

This study had several limitations. Our collection may not represent the prevalence and evolution of carbapenem-resistant *E. cloacae* in China. Our study lacks a comprehensive analysis of clinical risk factors. We were unable to determine complete genetic structures owing to the limitation of short-read sequencing. Long-read sequencing techniques would provide more detailed analysis of integron structures and plasmid backbones.

In conclusion, our study revealed that carbapenemase, ESBL and/or AmpC overexpression combined with porin loss were the primary mechanisms responsible for carbapenem resistance. Moreover, we initially reported the nosocomial outbreak caused by NDM-1-producing *E. cloacae* ST78 in Northwest China. Therefore, it emphases a critical concern to monitor and control the further spread of NDM-1 in China.

## Author Contributions

BH, LC, and Y-WT designed the study. YC, XY, KLa, MZ, XM, and YH carried out the experiments. CC, WJ, JZ, KLi, PG, and WZ analyzed the data. YC wrote the manuscript.

### Conflict of Interest Statement

The authors declare that the research was conducted in the absence of any commercial or financial relationships that could be construed as a potential conflict of interest.
